# Green Tea Catechin Is an Alternative Immune Checkpoint Inhibitor that Inhibits PD-L1 Expression and Lung Tumor Growth

**DOI:** 10.3390/molecules23082071

**Published:** 2018-08-18

**Authors:** Anchalee Rawangkan, Pattama Wongsirisin, Kozue Namiki, Keisuke Iida, Yasuhito Kobayashi, Yoshihiko Shimizu, Hirota Fujiki, Masami Suganuma

**Affiliations:** 1 Graduate School of Science and Engineering, Saitama University, Saitama 338-8570, Japan; ewmedsci@gmail.com (A.R.); wongsiri.patt@gmail.com (P.W.); k.namiki1080@gmail.com (K.N.); 2 Research Institute for Clinical Oncology, Saitama Cancer Center, Saitama 362-0806, Japan; 3 Molecular Chirality Research Center and Department of Chemistry, Graduate School of Science, Chiba University, Chiba 263-8522, Japan; kiida@chiba-u.jp; 4 Saitama Cardiovascular and Respiratory Center, Kumagaya, Saitama 360-0197, Japan; kobayashiyasuhito@yahoo.co.jp (Y.K.); shimizu.yoshihiko@pref.saitama.lg.jp (Y.S.); 5 Faculty of Medicine, Saga University, Saga 849-8501, Japan; uv4h-fjk@asahi-net.or.jp

**Keywords:** (−)-epigallocatechin gallate, immune checkpoint, interferon-γ, epidermal growth factor, lung tumor

## Abstract

The anticancer activity of immune checkpoint inhibitors is attracting attention in various clinical sites. Since green tea catechin has cancer-preventive activity in humans, whether green tea catechin supports the role of immune checkpoint inhibitors was studied. We here report that (−)-epigallocatechin gallate (EGCG) inhibited programmed cell death ligand 1 (PD-L1) expression in non–small-cell lung cancer cells, induced by both interferon (IFN)-γ and epidermal growth factor (EGF). The mRNA and protein levels of IFN-γ–induced PD-L1 were reduced 40–80% after pretreatment with EGCG and green tea extract (GTE) in A549 cells, via inhibition of JAK2/STAT1 signaling. Similarly, EGF-induced PD-L1 expression was reduced about 37–50% in EGCG-pretreated Lu99 cells through inhibition of EGF receptor/Akt signaling. Furthermore, 0.3% GTE in drinking water reduced the average number of tumors per mouse from 4.1 ± 0.5 to 2.6 ± 0.4 and the percentage of PD-L1 positive cells from 9.6% to 2.9%, a decrease of 70%, in lung tumors of A/J mice given a single intraperitoneal injection of 4-(methylnitrosamino)-1-(3-pyridyl)-1-butanone (NNK). In co-culture experiments using F10-OVA melanoma cells and tumor-specific CD3+ T cells, EGCG reduced *PD-L1* mRNA expression about 30% in F10-OVA cells and restored *interleukin-2* mRNA expression in tumor-specific CD3+ T cells. The results show that green tea catechin is an immune checkpoint inhibitor.

## 1. Introduction

Blockade of programmed cell death ligand 1 (PD-L1)/programmed cell death 1 (PD-1) immune checkpoints by monoclonal antibodies has shown measurable success in cancer therapy against a variety of tumor types, including non–small-cell lung cancer (NSCLC) [1,2]. PD-L1 is expressed on both tumor cells and immune cells, and PD-1 is predominantly expressed on activated T cells. Binding of PD-L1 to PD-1 inhibits T cell effector function by inducing exhaustion and apoptosis of T cells, resulting in an immunosuppressive state [3]. Expression of PD-L1 in tumor cells plays an important role in tumor immune escape and cancer progression. Although immunotherapy targeting PD-L1/PD-1 signaling is being used for treatment of advanced lung cancers, the benefit is limited to the early stages, because antibody-based checkpoint inhibitors are associated with unique immune-related adverse effects and high costs [4]. Recently, bromodomain and extraterminal (BET) inhibitors have also been shown to be inhibitors of PD-L1 expression by directly targeting the *PD-L1* gene [5]. Apigenin, a phytochemical, also inhibits interferon (IFN)-γ–induced PD-L1 protein [6]. Development of small-molecule blocking PD-L1/PD-1 signaling is now being actively investigated. 

Green tea and (−)-epigallocatechin gallate (EGCG), the main constituent of green tea catechins, are nontoxic, effective cancer preventives for humans [7]: drinking 10 cups (120 mL/cup) of green tea per day delayed cancer onset in a 10-year prospective cohort study in Japan, and also prevented colorectal adenoma recurrence in a double-blind randomized phase II clinical trials in Japan and Korea [7,8,9,10]. Recently we reported that human cancer stem cells (CSCs) are a target for cancer prevention using EGCG [7], based on evidence that EGCG generally inhibits the self-renewal of CSCs and the expression of epithelial-mesenchymal transition (EMT) phenotypes in human CSCs. Green tea catechins are tannins that can bind to various proteins and nucleic acids [11,12]. EGCG inhibits the binding of various ligands, tumor promoters, and epidermal growth factor (EGF) to their receptors in the cell membrane, which is called the “sealing effects” of EGCG. This is achieved by stiffening of the cell membrane after EGCG treatment [11]. Since EGCG inhibits metastasis of mouse B16 melanoma cells and enhances anticancer activity in combination with anticancer agents [13,14], we propose that EGCG may have additional clinical benefits through immunological interactions. The expression of PD-L1 on tumor cells is induced by EMT, IFN-γ, tumor necrosis factor-α (TNF-α), and EGF in the inflammatory tumor microenvironment [3,15,16]. Therefore, we hypothesize that EGCG will inhibit PD-L1, an immune checkpoint molecule, leading to enhancement of the antitumor immune response.

We first examined the effects of EGCG on PD-L1 expression induced by two factors, IFN-γ and EGF, in NSCLC cell lines in vitro. This is because IFN-γ is the strongest stimulator of PD-L1 expression, and EGF and EGF receptor (EGFR) mutations induce PD-L1 expression with lung cancer progression [1,2,16]. We then studied the relationship between inhibition of PD-L1 expression and lung tumor growth by giving water containing 0.3% green tea extract (GTE), a freeze-dried form of green tea infusion, to A/J mice treated with a tobacco-specific carcinogen, 4-(methylnitrosamino)-1-(3-pyridyl)-1-butanone (NNK), in vivo. In addition, to determine whether EGCG reverses the inhibitory effect of the PD-L1/PD-1 pathway on T cell activity, we conducted a co-culture experiment using F10-OVA mouse melanoma cells and tumor-specific CD3+ T cells isolated from the spleens of F10-OVA–immunized C57BL/6 mice.

In this study, we found that EGCG and GTE inhibited both IFN-γ– and EGF-induced PD-L1 expression by inhibiting two signaling pathways, JAK2/STAT1 and EGFR/Akt, in human NSCLC cell lines. In addition, oral administration of GTE reduced the percentage of PD-L1–positive cells in lung tumors and the average number of tumors per mouse in A/J mice treated with NNK. EGCG also reduced *PD-L1* mRNA expression in F10-OVA cells and partially restored *interleukin-2* (*IL-2*) mRNA expression in tumor-specific T cells in a co-culture experiment. This is the first report showing that EGCG and GTE have some activity as immune checkpoint inhibitors in lung cancer development.

## 2. Results

### 2.1. Downregulation of IFN-γ–Induced PD-L1 Protein and Inhibition of STAT1 and Akt Phosphorylation in A549 Cells Treated with GTE and EGCG

**Figure 1:**
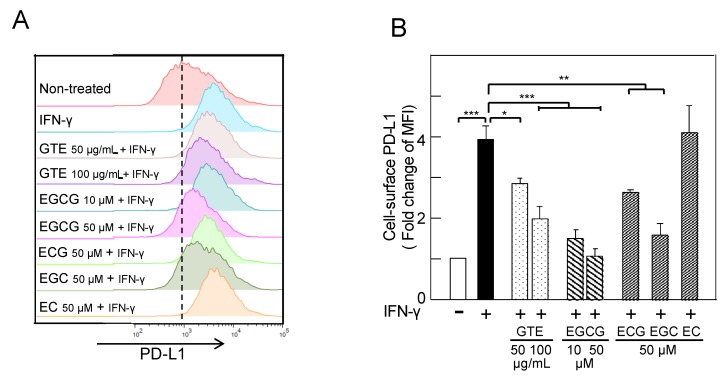
Inhibition of interferon (IFN)-γ–induced cell-surface programmed cell death ligand 1 (PD-L1) protein by green tea extract (GTE) and green tea catechins in A549 cells. (**A**) Cell-surface PD-L1, and (**B**) average of fold change of median fluorescence intensity (MFI). “−“ and “+” indicate in the absence or presence of IFN-γ (10 ng/mL). * *p* < 0.05, ** *p* < 0.01, *** *p* < 0.001. EGCG, (−)-epigallocatechin gallate; ECG, (−)-epicatechin gallate; EGC, (−)-epigallocatechin; EC, (−)-epicatechin.

**Figure 2:**
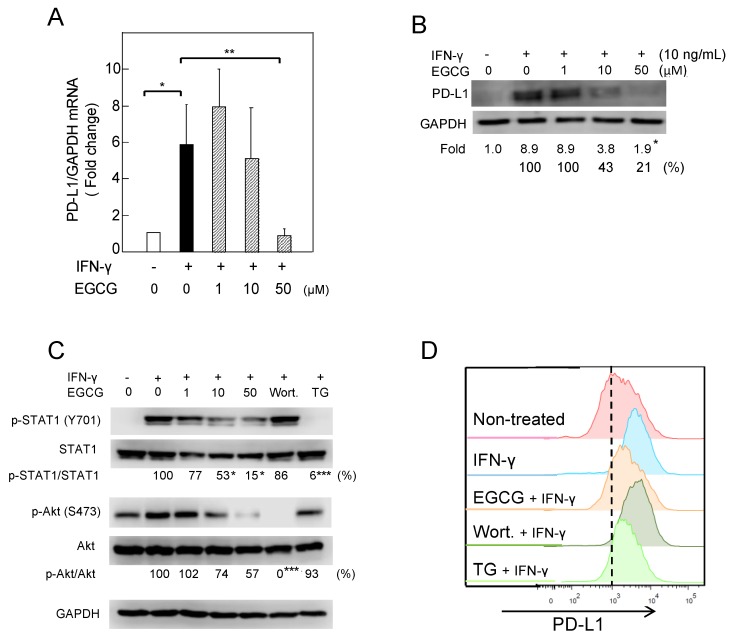
Downregulation of IFN-γ–induced PD-L1 protein and inhibition of STAT1- and Akt-phosphorylation in A549 cells treated with (−)-epigallocatechin gallate (EGCG). (**A**) *PD-L1* mRNA expression, (**B**) PD-L1 protein, (**C**) phosphorylation of STAT1 and Akt, and (**D**) cell-surface PD-L1. “−“ and “+” indicate in the absence or presence of IFN-γ (10 ng/mL). Numbers indicate average percentage compared with IFN-γ–treated cells. * *p* < 0.05, ** *p* < 0.01, *** *p* < 0.001. GAPDH, glyceraldehyde-3-phosphate dehydrogenase.

It is well known that IFN-γ, produced by activated T cells, stimulates PD-L1 expression in the tumor microenvironment [1,2], so we first examined the effects of green tea catechins on IFN-γ–induced PD-L1 expression. Treatment with IFN-γ (10 ng/mL) increased the mRNA and protein of PD-L1 and cell-surface PD-L1 protein twofold in A549 cells (Supplementary Figure S1). GTE contains at least four catechins: EGCG, (−)-epicatechin gallate (ECG), (−)-epigallocatechin (EGC), and (−)-epicatechin (EC) [17]. Pretreatment of A549 cells with 50 or 100 µg/mL GTE reduced the levels of cell-surface PD-L1 protein induced by IFN-γ (Figure 1A); cells pretreated with EGCG (10 and 50 µM), ECG (50 µM), or EGC (50 µM) reduced PD-L1 protein levels from 3.8 ± 0.3 median fluorescence intensity (MFI) to 2.8 ± 0.1 to 1.0 ± 0.2 MFI, a decrease of 40–80%. EC, an inactive catechin, had no effect on PD-L1 protein expression. EGCG showed the most potent inhibition among the green tea catechins (Figure 1B). Pretreatment of the cells with EGCG dose-dependently inhibited *PD-L1* mRNA and protein, and 50 µM EGCG decreased *PD-L1* mRNA by 86% (from 5.8-fold to 0.8-fold) and PD-L1 protein by 79% (Figure 2A,B). A similar reduction of IFN-γ–induced PD-L1 expression with EGCG was observed in H1299 cells (Supplementary Figure S2). 

To clarify the inhibitory mechanisms of EGCG, we next studied the IFN receptor (IFNR) signaling pathway. Pretreatment with EGCG dose-dependently reduced the levels of p-STAT1 and p-Akt: 50 µM EGCG inhibited p-STAT1 and p-Akt by 85% and 43%, respectively, in A549 cells (Figure 2C). Furthermore, pretreatment of A549 with 1 µM TG-101348 (TG), a JAK2 inhibitor, reduced p-STAT1 by 94%, but did not affect p-Akt, and showed a strong reduction of cell-surface PD-L1 protein, as EGCG did (Figure 2C,D). However, wortmannin, a phosphoinositide 3-kinase (PI3K) inhibitor, inhibited p-Akt, but did not show any reduction of cell-surface PD-L1 (Figure 2C,D). These results indicate that EGCG reduced PD-L1 expression via inhibition of the JAK2/STAT1 signaling pathway. 

### 2.2. Downregulation of EGF-Induced PD-L1 Protein and Inhibition of Akt Phosphorylation in Lu99 Cells Treated with EGCG

**Figure 3:**
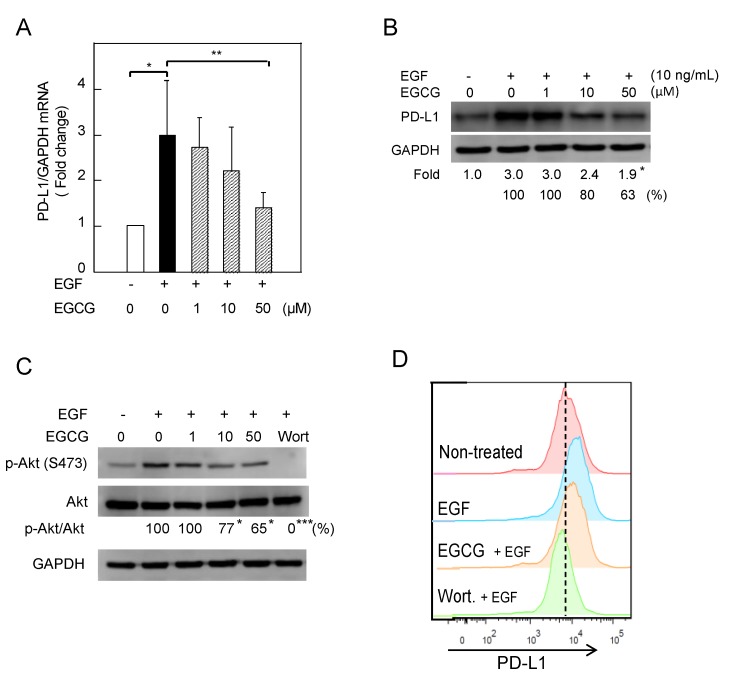
Downregulation of EGF-induced PD-L1 protein and inhibition of Akt phosphorylation in Lu99 cells treated with EGCG. (**A**) *PD-L1* mRNA expression, (**B**) PD-L1 protein, (**C**) phosphorylation of STAT1 and Akt, and (**D**) cell-surface PD-L1. “−“ and “+” indicate in the absence or presence of EGF (10 ng/mL). Numbers indicate average percentage compared with EGF-treated cells. * *p* < 0.05, ** *p* < 0.01, *** *p* < 0.001.

Activation of EGFR signaling by EGF and EGFR mutations also drove PD-L1 expression in NSCLC cells [16,18]. Treatment with 10 ng/mL EGF significantly increased *PD-L1* mRNA and protein expression 5.8-fold and 8.9-fold, respectively, and cell-surface PD-L1 about 2.7-fold in Lu99 cells (Supplementary Figure S1). Pretreatment with 50 µM EGCG for 3 h decreased the levels of *PD-L1* mRNA and protein in Lu99 cells by 50% and 37%, respectively (Figure 3A,B). It is important to note that EGCG inhibited the EGFR signaling pathway. Pretreatment with 50 µM EGCG decreased p-Akt by 35%, and pretreatment with 1 µM wortmannin reduced p-Akt and cell-surface PD-L1 levels more than EGCG did (Figure 3C,D). Although the effects of EGCG on the EGFR/Akt axis and cell-surface PD-L1 were not strong, EGCG inhibited the production of EGF-induced PD-L1. Overall, EGCG inhibited PD-L1 expression in NSCLC cells induced by two different factors via their specific receptors. 

### 2.3. Oral Administration of GTE Reduced PD-L1–Positive Cells and Inhibited Tumor Growth in the Lungs of NNK-Treated A/J Mice

**Figure 4:**
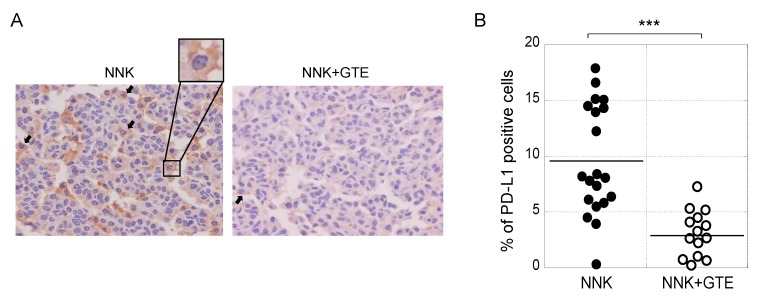
Oral administration of GTE reduced PD-L1–positive cells and inhibited tumor development in the lungs of NNK-treated A/J mice. (**A**) Representative immunohistochemical staining with anti–PD-L1 antibody. Black arrows indicate PD-L1–positive cells on the plasma membrane. (**B**) Average percentage of PD-L1–positive cells in individual tumors. *** *p* < 0.001.

Next, we conducted lung carcinogenesis experiments with female A/J mice. A single intraperitoneal injection of NNK produced lung tumors in 100% of the mice after 16 weeks. Oral administration of 0.3% GTE in drinking water reduced the average number of tumors per mouse from 4.1 ± 0.5 to 2.6 ± 0.4 at week 16, a decrease of 37% (Table 1). All tumors were adenomas 0.8 mm or more in diameter. Immunohistochemical analysis with anti–PD-L1 antibody showed PD-L1 protein on the plasma membrane and in the cytosol of lung tumor cells; cells with PD-L1 on the plasma membrane were counted as PD-L1–positive cells (Figure 4A). Figure 4B shows average percentage of PD-L1–positive cells in individual tumors in the NNK and NNK + GTE groups. The NNK group had an average of 9.6 ± 4.9% PD-L1–positive cells, while the NNK + GTE group had 2.9 ± 2.2%, a decrease of 70% (Figure 4B and Table 1). GTE significantly reduced PD-L1 protein in lung tumors in vivo, which was associated with inhibition of tumor development. It is important to note that a solution of 0.3% (3 g/L) GTE contains 0.85 g/L of catechins (14% EGCG, 8% ECG, 3% EGC, and 3.5% EC) and 0.1 g/L of caffeine, corresponding to the totals in green tea beverages in Japan [19].

### 2.4. EGCG Slightly Restored IL-2 mRNA Expression in Tumor-Specific CD3+ T Cells Co-cultured with Tumor Cells (F10-OVA)

**Figure 5:**
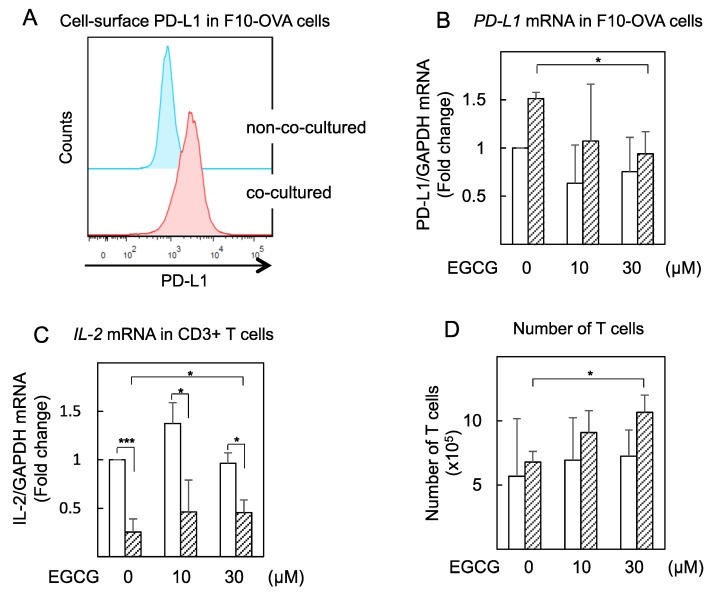
EGCG reduced *PD-L1* mRNA expression in co-cultured F10-OVA cells and restored *IL-2* mRNA expression in co-cultured tumor-specific CD3+ T cells. (**A**) Cell-surface PD-L1 and (**B**) *PD-L1* mRNA in F10-OVA cells alone (open bars) and cells co-cultured with CD3+ T cells (shaded bars). mRNA expression normalized by GAPDH in nontreated cells is expressed as 1 in (**B**) and (**C**). (**C**) *IL-2* mRNA in CD3+ T cells alone (open bars) and cells co-cultured with F10-OVA cells (shaded bars). (**D**) Number of T cells in CD3+ T cells alone (open bars) and cells co-cultured with F10-OVA cells (shaded bars) were counted by trypan blue exclusion method. * *p* < 0.05, *** *p* < 0.001.

We conducted a co-culture experiment using B16-F10 mouse melanoma cells expressing ovalbumin (F10-OVA) and tumor-specific mouse CD3+ T cells isolated from the spleens of F10-OVA-immunized C57BL/6 mice. The cell-surface *PD-L1* in F10-OVA cells increased twofold after co-culture with tumor-specific CD3+ T cells compared with F10-OVA cells alone (Figure 5A). Next, we found that EGCG (30 μM) reduced *PD-L1* mRNA expression approximately 30% in F10-OVA cells co-cultured with CD3+ T cells, but EGCG did not affect *PD-L1* mRNA level in F10-OVA cells not co-cultured (Figure 5B). 

In addition, we measured *IL-2* mRNA expression in CD3+ T cells to determine T cell effector activity. After co-culture with F10-OVA cells for 48 h, *IL-2* mRNA expression was dramatically decreased by 24%. Treatment with EGCG (10 μM) recovered *IL-2* mRNA to approximately 40% (Figure 5C) and increased the number of T cells by 1.6-fold (Figure 5D) in CD3+ T cells co-cultured with F10-OVA cells, although EGCG did not affect *IL-2* mRNA expression or number of T cells in non–co-cultured CD3+ T cells. These results indicate that EGCG partially restored T cell activity by suppressing PD-L1/PD-1 signaling.

## 3. Discussion

We showed, for the first time, that the main green tea catechin, EGCG, acts as an immune checkpoint inhibitor by inhibiting PD-L1 expression in tumor cells. For example, oral administration of GTE, which corresponds to green tea beverages consumed by Japanese people every day, reduced PD-L1 expression in lung tumors and the average number of tumors per mouse in A/J mice treated with NNK, so EGCG and GTE can probably reduce PD-L1 expression. In addition, results of co-culture experiments with F10-OVA tumor cells and tumor-specific CD3+ T cells support our conclusion that EGCG-mediated PD-L1 inhibition results in restoration of T cell activity. 

Interestingly, EGCG inhibited both IFNR/JAK2/STAT1 and EGFR/Akt signaling pathways, suggesting that EGCG inhibits IFN-γ-IFNR and EGF-EGFR activation by inhibiting ligand-receptor binding [11]. The results are consistent with a recent report that EGCG increased the bending stiffness of artificial lipid membranes by adsorption of galloyl catechin aggregates to the lipid membrane surface [20]. We also reported that stiffening of cancer cell membranes with EGCG correlates well with inhibition of EMT, motility, and metastasis in lung cancer cells and B16-F10 mouse melanoma cells [11,21]. It is notable that Gimzewski’s group reported that GTE increased cell stiffness of tumor cells isolated from the pleural effusions of various cancer patients, but it had no effect on normal mesothelial cells [22]. It is well known that membrane lipids such as cholesterol regulate T cell signaling and function [23]; whether EGCG directly enhances T-cell function or acts indirectly will require further study. In addition to our results, EGCG was previously shown to suppress indoleamine 2,3-dioxygenase (IDO), which can enhance immune escape by blocking the IFN-γ–induced JAK/PKC/STAT1 signaling pathway in oral cancer cells [24]. These results strongly indicate that enhancing the effects of EGCG on adaptive immune cells will restrict the growth of tumor cells. 

Based on results showing that the expression of *PD-L1* gene and cell-surface PD-L1 protein varied depending on the inducer and the cells, as shown in Supplementary Figure S1, we stimulated A549 cells with IFN-γ and Lu99 cells with EGF to induce PD-L1 expression. It is well known that PD-L1 protein in lung cancer cells is induced by activation of EGFR signaling through both overexpression and mutation of EGFR [16]. Lu99 cells showed high intrinsic PD-L1 levels and a strong response to EGF among the three lung cancer cell lines studied. This is probably because of high EGFR protein levels and a mutated T1025A in PI3K catalytic subunit α (PI3KCA) [25]. It has recently been reported that multiple microRNAs act as important regulators of PD-L1 expression directly or indirectly [26]. Since EGCG upregulates tumor suppressor microRNAs, it is important to determine whether alteration of microRNA levels regulated by EGCG inhibits *PD-L1* mRNA expression [27].

Phase III clinical trials recently reported that the combination of chemotherapy and immune checkpoint–targeted antibodies shows an effect superior to chemotherapy alone, indicating that the use of immune checkpoint inhibitors will be extended to cancer treatment [4]. We previously reported that the combination of EGCG and anticancer compounds showed synergistic enhancement of anticancer effects in numerous human cancer cell lines and xenograft mouse models, in vitro and in vivo [14,28]. Since EGCG and GTE act as immune checkpoint inhibitors, we think the combination of green tea catechins and checkpoint inhibitors will further increase the benefits of cancer therapy.

It is important to note that PD-L1 in tumor cells has functions other than as an immune checkpoint ligand, including stimulation of cancer progression, promotion of EMT, acquisition of tumor-initiating potential, and resistance to apoptosis [15]. As our previous experiments showed, EGCG and GTE inhibit EMT in lung cancer cells, increase cell stiffening, and inhibit self-renewal of cancer stem cells, leading to apoptosis of cancer cells [7,11,21].

## 4. Materials and Methods

### 4.1. Cell Lines and Chemicals

Human NSCLC cell lines A549, H1299 (American Type Culture Collection, Manassas, VA, USA), and Lu99 (Riken Bioresource Center, Tsukuba, Ibaraki, Japan) were cultured in RPMI 1640 supplemented with 10% fetal bovine serum (FBS) (Sigma-Aldrich, St. Louis, MO, USA). Mouse B16-F10 melanoma were kindly provided by Dr. Shun’ichiro Taniguchi at Shinshu University, Japan. EGCG (more than 99% purity) was purified from Japanese green tea leaves (*Camellia sinensis* L., O. Kuntze, Theaceae), and GTE was extracted by a similar procedure to make green tea infusion (sencha) as described previously [17,19]. Green tea leaves were cultivated at Saitama Prefectural Tea Institute, Saitama, Japan, and processed to make sencha. After brewing 2 kg of green tea leaves (sencha) in 70 L of hot water (85 °C) for 15 min, the green tea infusion was filtrated and freeze-dried. About 500 g of GTE was obtained. This GTE contained 14% EGCG, 8% ECG, 3% EGC, 3.5% EC, and 3.3% caffeine as analyzed by HPLC. ECG (>99%), EGC (>99%), and EC (>99%) were purchased from Funakoshi Co. Ltd., Tokyo, Japan. Anti-PD-L1 (Abcam, Cambridge, MA, USA ), anti-STAT1, anti-phospho-STAT1 (BD Bioscience, NJ, USA), anti-Akt, anti-phospho-Akt (Cell Signaling Technology, Danvers, MA, USA) and anti- glyceraldehyde-3-phosphate dehydrogenase (GAPDH) (Trevigen, Gaithersburg, MD, USA) antibodies were used for the experiments. Recombinant human IFN-γ and human EGF were obtained from R&D Systems (Minneapolis, MN, USA) and PeproTech (Rocky Hill, London, UK), respectively. Wortmannin and TG-101348 were purchased from Sigma-Aldrich (St. Louis, MO, USA), and ChemScene (Monmouth Junction, NJ, USA), respectively.

### 4.2. Animals

Female A/J mice and C57BL/6 mice were obtained from Japan SLC Inc. (Hamamatsu, Japan) and Charles River Laboratories Japan Inc. (Yokohama, Japan), respectively. The animal experiments were performed in accordance with protocols approved by the Institutional Animal Care and Use Committee of the Research Institute for Clinical Oncology, Saitama Cancer Center (project identification code: 21-1) and the Saitama University Committee on Animal Research (project identification code: H29-A-1-12), under Fundamental Guidelines for Proper Conduct of Animal Experiments and Related Activities in Academic Research Institutions and Acts on Welfare and Management of Animals. All mice were housed at 23 ± 2 °C with a 12 h light-dark cycle. Mice were fed food and water *ad libitum*.

### 4.3. Establishment of Ovalbumin-Expressing B16-F10 (F10-OVA) Cells

F10-OVA cells were established by transfection of pcDNA3-OVA plasmid (Addgene, Cambridge, MA, USA) using Lipofectamine^®^ 3000 Transfection reagent (Invitrogen, Thermo Fisher Scientific, Waltham, MA, CA, USA) to B16-F10 cells. F10-OVA cells were maintained with 2 mg/mL G418 sulfate in Dulbecco's Modified Eagle's medium (DMEM) containing 10% FBS. The F10-OVA clones were confirmed by ovalbumin gene expression using PCR.

### 4.4. Quantitative RT-PCR 

Total RNA of the cells was extracted using ISOGEN (Nippon Gene Co. Ltd., Toyama, Japan). cDNA was synthesized from total RNA using Oligo(dT)_16_ and MuLV reverse transcriptase (Thermo Fisher Scientific, Cambridge, MA, USA), and real-time PCR was conducted using SYBR Green I (LightCycler 480, Roche Lifescience, Basel, Switzerland), as described previously [29]. Primers used were as follows:
human PD-L1 forward primer5′-GGACAAGCAGTGACCATCAAG-3′human PD-L1 reverse primer5′-CCCAGAATTTACCAAAGTGAGTCCT-3′human GAPDH forward primer5′-TGGTATCGTGGAAGGACTCATGAC-3′human GAPDH reverse primer5′-ATGCCACTCAGCTTCCCGTTCAGC-3′mouse PD-L1 forward primer5′-GGACAAGCAGTGACCATCAAG-3′mouse PD-L1 reverse primer5′-TGATCTGAAGGGCAGCATTTC-3′mouse IL-2 forward primer5′-TTGTCGTCCTTGTCAACAGC-3′mouse IL-2 reverse primer5′-CTGGGGAGTTTCAGGTTCCT-3′mouse GAPDH forward primer5′-T TTGTCGTCCTTGTCAACAGC-3′mouse GAPDH reverse primer5′-CTGGGGAGTTTCAGGTTCCT-3′

The *PD-L1 and IL-2* mRNA relative expressions were normalized by *glyceraldehyde-3-phosphate dehydrogenase (GAPDH)* mRNA expression as an internal control. Results were obtained from at least 3 independent experiments.

### 4.5. Western Blot Analysis

Whole cell lysates were obtained with radioimmunoprecipitation assay (RIPA) buffer containing 50 mM Tris-HCl (pH 7.4), 1% NP-40, 0.5% sodium deoxycholate, 0.1% sodium dodecyl sulfate (SDS), 150 mM sodium chloride, 2 mM ethylenediaminetetraacetic acid (EDTA), 10 μg/mL aprotinin, 10 μg/mL leupeptin, 1 mM phenylmethanesulfonyl fluoride, 2.5 mM sodium pyrophosphate, 1 mM sodium orthovanadate, and 2.5 mM sodium fluoride. Cell lysates were subjected to gel electrophoresis and then transferred onto a nitrocellulose membrane. After incubation with the primary antibody (1:1000) followed by an appropriate secondary antibody (1:2000), specific bands were detected by Immunostar LD (Wako Pure Chem. Japan Ind. Ltd., Osaka, Japan), using C-DiGit Chemiluminescent Western Blot Scanner (LI-COR Biosciences Inc., Lincoln, NE, USA) [29]. GAPDH was used as an internal control. The values are the average fold changes for nontreated cells obtained from at least 3 independent experiments. 

### 4.6. Flow Cytometry

Cells were stained with anti–PD-L1 antibody (1:200) in phosphate-buffered saline (PBS) with 2% FBS and 0.02% EDTA for 30 min, and then incubated with Alexa Fluor^®^ 488 goat anti-rabbit IgG (Invitrogen, Waltham, MA, USA) for 20 min on ice. Labeled cells were analyzed by flow cytometry (FACSCanto II, BD Biosciences, San Jose, CA, USA). The data were analyzed by FlowJo v.10 software (FlowJo, LLC, Ashland, OR, USA) and the levels of cell-surface PD-L1 protein were estimated by median fluorescence intensity (MFI). The experiments were performed at least 3 times.

### 4.7. Development of Lung Tumors

Female A/J mice 7 weeks old were given a single intraperitoneal injection of NNK (100 mg/kg body weight; Toronto Research Chemicals Inc., North York, ON, Canada), as described previously [28,30]. Two days later, 15 mice received drinking water containing 0.3% GTE and 20 mice received drinking water without GTE for 16 weeks. After sacrifice, the lungs were kept and tumor size was measured. Lung tumors measuring 0.8 mm or more in diameter were counted. 

### 4.8. Immunohistochemical Staining

Lung sections were incubated with 3% H_2_O_2_ for 10 min and then subjected to antigen retrieval using pressure cooking in antigen-activating solution (pH 9, 98 °C). After blocking with Protein Block (Dako, Carpinteria, CA, USA) for 5 min at room temperature, the sections were immunostained with anti–PD-L1 antibody (1:200) for 40 min at 37 °C, followed by secondary antibody (N-Histofine Simple Stain MAX-PO (Multi), Nichirei Bioscience Inc., Tokyo, Japan) for 20 min at 37 °C, as described previously [31]. Cells showing positive for PD-L1 on the plasma membrane were independently counted by 3 investigators. Results are expressed as average percentage of PD-L1–positive cells ± standard error (SE).

### 4.9. Co-culture with Tumor-Specific CD3+ T Cells and F10-OVA Cells

Tumor-specific CD3+ T cells were generated as previously described [32]. Briefly, F10-OVA cells were treated with 25 µg/mL of mitomycin C in DMEM containing 10% FBS for 20 min at 37 °C in the dark, and washed with PBS. C57BL/6 mice were intraperitoneally injected with mitomycin C–treated F10-OVA cells, and 14 days later CD3+ T cells were isolated from the spleen using anti-CD3 antibody-coated magnetic microbeads (Miltenyi Biotec, GmbH, Bergisch Gladbach, Germany). Mitomycin C–treated F10-OVA cells were pretreated with EGCG for 3 h, and then co-cultured with CD3+ T cells at a 1:20 ratio in the presence of EGCG for 48 h. 

### 4.10. Statistical Analysis

Statistical analyses were performed using one-way analysis of variance (ANOVA) followed by Dunnett’s test. Each experiment was conducted independently at least three times, and values are expressed as mean ± standard deviation (SD). For the in vivo lung carcinogenesis experiment, Student’s *t*-test and Wilcoxon–Mann–Whitney test were used, and values are expressed as mean ± SE. *p* value < 0.05 was considered statistically significant.

## 5. Conclusions

All our results suggest that EGCG partially restores T cell activity by inhibition of PD-L1/PD-1 signaling, resulting in inhibition of lung cancer growth. Thus, we present a new concept: green tea catechin acts as an immune checkpoint inhibitor, leading to cancer prevention and treatment.

## Figures and Tables

**Table 1:** Oral administration of GTE reduced average number of lung tumors and percentage of PD-L1–positive cells in 4-(methylnitrosamino)-1-(3-pyridyl)-1-butanone (NNK)-treated A/J mice.

Group	Average No. ofTumors/Mouse ± SE (% of inhibition)	Percentage of PD-L1–Positive Cells ± SE (% of inhibition)
NNK	4.1 ± 0.5	9.6 ± 4.9
NNK + GTE	2.6 ± 0.4 (36.6) *	2.9 ± 2.2 (69.8) *

* *p* < 0.05.

## Supplementary Materials

Supplementary Figures S1 and S2 are available online.
