# Supplementary material for: Green Tea Catechin Is an Alternative Immune Checkpoint Inhibitor that Inhibits PD-L1 Expression and Lung Tumor Growth
Source: Molecules. 2018 Aug 18;23(8):2071. doi: 10.3390/molecules23082071 (PMC6222340; doi:10.3390/molecules23082071)
Supplement: Supplementary file 1 [file molecules-23-02071-s001.pdf]

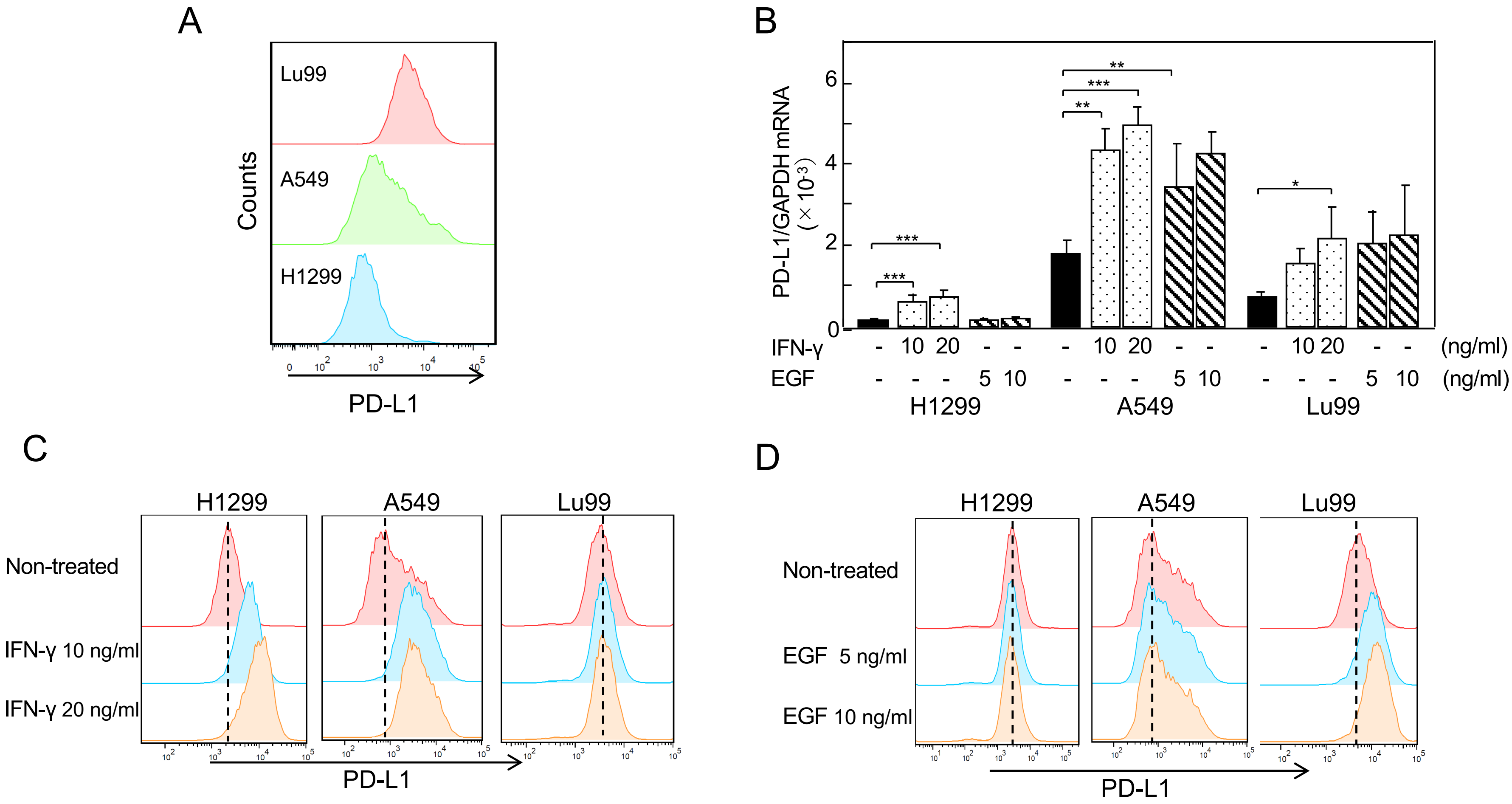

Figure S1. Expression of *PD-L1* gene and cell-surface PD-L1 protein in NSCLC cells induced by IFN-γ or EGF. (A) Intrinsic cell-surface PD-L1 protein, (B) Expression of *PD-L1* mRNA by treatment with IFN-γ or, (C) Cell-surface PD-L1 in IFN-γ- treated cells, (D) Cell-surface PD-L1 in EGF-treated-cells. *$p < 0.05$, **$p < 0.01$, ***$p < 0.001$

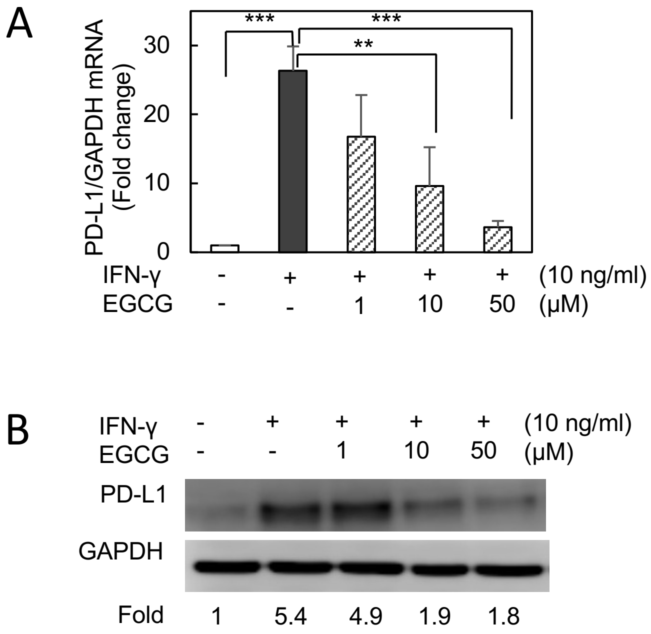

Figure S2. Down-regulation of IFN-γ-induced *PD-L1* mRNA and protein in H1299 cells by EGCG. (A) *PD-L1* mRNA, and (B) PD-L1 protein. Numbers indicate average fold-expression compared with non-treated cells. **$p < 0.01$, ***$p < 0.001$
